# Antimicrobial resistance of *Pseudomonas* spp. isolated from wastewater and wastewater-impacted marine coastal zone

**DOI:** 10.1007/s11356-015-5098-y

**Published:** 2015-08-19

**Authors:** Aneta Luczkiewicz, Ewa Kotlarska, Wojciech Artichowicz, Katarzyna Tarasewicz, Sylwia Fudala-Ksiazek

**Affiliations:** Department of Water and Wastewater Technology, Faculty of Civil and Environmental Engineering, Gdansk University of Technology, Narutowicza 11/12, 80-233 Gdansk, Poland; Institute of Oceanology Polish Academy of Sciences, Powstancow Warszawy 55, 81-712 Sopot, Poland

**Keywords:** *Pseudomonas* spp., Species distribution, Antimicrobial susceptibility, Wastewater, Marine outfall

## Abstract

In this study, species distribution and antimicrobial susceptibility of cultivated *Pseudomonas* spp. were studied in influent (INF), effluent (EFF), and marine outfall (MOut) of wastewater treatment plant (WWTP). The susceptibility was tested against 8 antimicrobial classes, active against *Pseudomonas* spp.: aminoglycosides, carbapenems, broad-spectrum cephalosporins from the 3rd and 4th generation, extended-spectrum penicillins, as well as their combination with the β-lactamase inhibitors, monobactams, fluoroquinolones, and polymyxins. Among identified species, resistance to all antimicrobials but colistin was shown by *Pseudomonas putida*, the predominant species in all sampling points. In other species, resistance was observed mainly against ceftazidime, ticarcillin, ticarcillin-clavulanate, and aztreonam, although some isolates of *Pseudomonas aeruginosa*, *Pseudomonas fluorescens*, *Pseudomonas pseudoalcaligenes*, and *Pseudomonas protegens* showed multidrug-resistance (MDR) phenotype. Among *P. putida*, resistance to β-lactams and to fluoroquinolones as well as multidrug resistance become more prevalent after wastewater treatment, but the resistance rate decreased in marine water samples. Obtained data, however, suggests that *Pseudomonas* spp. are equipped or are able to acquire a wide range of antibiotic resistance mechanisms, and thus should be monitored as possible source of resistance genes.

## Introduction

The nonfermenting Gram-negative *Pseudomonas* genus was first described and classified in 1894 by Emil (Walter) Migula, a Poland-born German botanist (Palleroni 2003), and up till now more than 100 species have been distinguished (Peix et al. 2009). Due to the fast growth and adaptability to versatile environmental conditions (oxidative, nutritional, and other stresses), members of *Pseudomonas* are regarded as one of the most diverse and ubiquitous group, isolated from the variety of natural (e.g., soil, waters), clinical, and artificial niches (e.g., catheters, contact lenses, activated sludge). An important feature of *Pseudomonas* spp. is the production of a wide variety of extracellular products. Several functions have been attributed to extracellular polymeric substances (e.g., proteins, polysaccharides, enzymes, extracellular DNA), which are mainly involved in attachment processes, biofilm formation, and virulence (van Delden 2004). Additionally, biosurfactants secreted by *Pseudomonas* spp. increase the solubilization of hydrocarbons from non-aqueous phase liquids and make them available for microbial degradation. Thus, *Pseudomonas* spp. are able to mineralize numerous organic compounds (aromatic hydrocarbons, chloro- and nitro-organic compounds, pesticides, herbicides) and play a crucial role in the bioremediation and detoxification of contaminated sites. *Pseudomonas* spp. are also important members of microbial community active in wastewater treatment processes. They harbor the genes required for the synthesis (polyphosphate kinase—*ppk*) and degradation (exopolyphosphatase—*ppx*) of polyphosphate (polyP) (Tobin et al. 2007) as well as gene clusters associated with denitrification. The ability of *Pseudomonas* spp. to adapt and to grow under limited O_2_ is also of clinical importance, since the efficacy of many antimicrobial agents is reduced in anoxic condition (Line et al. 2014). It may explain, for example, the limited antimicrobial susceptibility of *Pseudomonas aeruginosa* in patients with cystic fibrosis. Despite respiratory tract infections, *P. aeruginosa* is also associated with urinary, gastrointestinal, soft tissue, bone, joint, and surgical site infections (EARS-Net. 2013).

In the USA, among 51,000 healthcare-associated infections caused by *P. aeruginosa*, 6000 (13 %) is multidrug-resistant, and up to 440 cases per year result in death (CDC 2013). Interestingly, *P. aeruginosa* is rarely linked with community-acquired infections but was recognized as one of the most common hospital pathogen, especially associated with the immunocompromised and the immunocompetent hosts (Livermore 2002). Other members of *Pseudomonas* spp. are also regarded as pathogens or opportunistic pathogens. They may infect plants (e.g., *Pseudomonas syringae*, *Pseudomonas savastanoi*, and *Pseudomonas pseudoalcaligenes*) as well as humans and animals (e.g., *P. aeruginosa*, *Pseudomonas fluorescens*, *Pseudomonas putida*, *Pseudomonas stutzeri*, *Pseudomonas anguilliseptica*) (Peix et al. 2009). Treatment of acute and chronic *Pseudomonas* infections is often ineffective. Besides biofilm formation and the ability to grow under anoxic conditions, *Pseudomonas* spp. show also limited susceptibility to many antimicrobials and disinfectants. Among *Pseudomonas* spp., the natural (intrinsic) resistance is mainly conferred by co-occurring mechanisms, such as low outer membrane permeability, β-lactamases synthesis, and the efflux systems. However, due to the remarkable plasticity of the genome, the members of *Pseudomonas* spp. are suspected to be able to acquire almost all known antimicrobial-resistance mechanisms (Livermore 2002). Thus, *Pseudomonas* infections are often associated with multi-drug resistance with limited treatment options.

Several studies have addressed the spread and susceptibility of *Pseudomonas* spp. in hospital settings, but little is known about their fate outside the hospital units. In this study, members of the *Pseudomonas* genus were isolated from the influent and effluent of a wastewater treatment plant (WWTP), as well as from its marine outfall. Special attention was given to the antimicrobial-resistance patterns carried by isolates of wastewater and marine water origin. Thus, the susceptibility was tested against antimicrobial classes that remain active against *Pseudomonas* spp.: aminoglycosides (e.g., amikacin, gentamicin, and tobramycin), carbapenems (imipenem and meropenem), broad-spectrum cephalosporins from the 3rd and 4th generation (ceftazidime and cefepime, respectively), extended-spectrum penicillins (piperacillin, ticarcillin) and their combination with the β-lactamase inhibitors (piperacillin–tazobactam and ticarcillin-clavulanate), monobactam (aztreonam), 2nd-generation fluoroquinolone (ciprofloxacin), and polymyxins (colistin). Isolates showing resistance to three or more of tested antimicrobial classes, except extended-spectrum penicillins, were defined as multidrug resistant (MDR). Statistical analyses were used to determine the dependencies between antimicrobial resistance patterns of tested *Pseudomonas* spp. Significant dependencies in antibiotic resistance prevalence and sampling point were also tested.

## Materials and methods

### Sampling

**Fig. 1:**
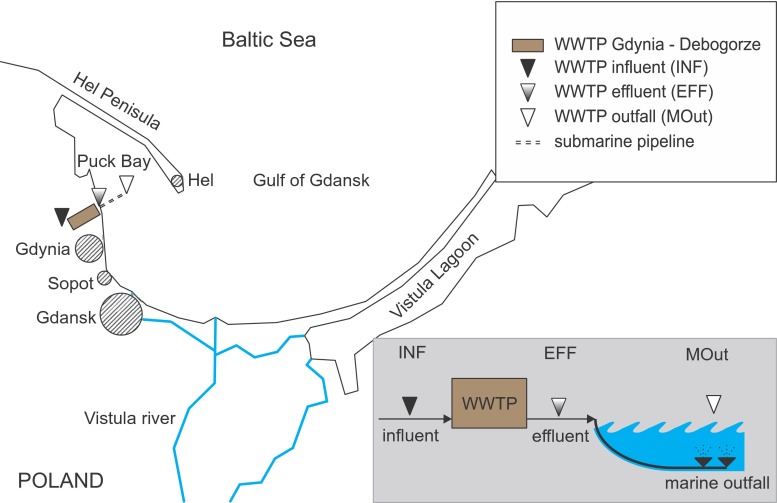
Sampling points: influent (*INF*), effluent (*EFF*) and marine outfall (*MOut*) of WWTP

The presence of *Pseudomonas* spp. was tested in wastewater received and discharged by WWTP into the Puck Bay—a shallow, western part of the Gulf of Gdansk, Baltic Sea, northern Poland (Fig. 1). During the sampling period, the tested WWTP received the municipal (~90 %), industrial (~10 %), and non-disinfected hospital wastewater (~0.2 %) with a pollutant load corresponding to 420,000 PE and an average flow rate of *Q*av. = 55,000 m^3^/day. The WWTP works in five (anaerobic, anoxic, aerobic, anoxic, re-aeration) zones Bardenpho system for nutrient removal, without final disinfection before discharging to marine waters.

In this study, 24-h flow proportionate samples of wastewater were taken after mechanical treatment (INF—influent) and after secondary clarifier (EFF—effluent). Additionally, the grab samples of marine water (MOut) were gathered in the Puck Bay, 2.4 km from the shore line (54° 37′ 08.4″ N, 18° 33′ 28.8″ E). In this location, treated wastewater is being discharged via submarine pipeline with diffusers systems since 2011 (Fig. 1).

All samples were taken once a month in April, June, August, and October in 2011, transported to the laboratory in the cooler box, and analyzed within 8 hours of collection.

### Basic physical and chemical analyses

Basic physical (pH) and chemical analyses (total nitrogen—TN, total phosphorus—TP, total suspended solids—TSS, chemical (COD), and biological oxygen demand (BOD_5_)) of tested wastewater and marine water samples were conducted according to APHA (2005).

### Isolation of culturable *Pseudomonas* spp.

The number of culturable *Pseudomonas* spp. was estimated using appropriate dilutions of analyzed samples (10^−1^–10^−3^ for wastewater and up to 100 cm^3^ for marine water samples) filtered in duplicate through 0.45-μm cellulose-acetate filters (Millipore, USA) and then placed on a *Pseudomonas* Isolation Agar (PIA, BD Diagnostic Systems, USA) and incubated at 37 °C. Typical *Pseudomonas* colonies appearing blue-green on PIA agar plates were enumerated as total *Pseudomonas* counts (TPC). All presumptive *Pseudomonas* were isolated from agar plates with less than 20 colonies, purified, and then stored in nutrient broth (Merck, Germany) supplemented with 15 % glycerol (Merck, Germany) at −80 °C. Overall, 146 isolates of presumptive *Pseudomonas* spp. (29 from INF, 81 from EFF, and 36 from MOut) were isolated and analyzed in this study.

### Identification and antimicrobial susceptibility testing of *Pseudomonas* spp.

In this study, the Phoenix Automated Microbiology System (BD Diagnostic Systems, USA) provided identification (ID) and susceptibility testing (AST) on commercially obtained Phoenix ID/AST panels, which included an ID side with dried substrates for bacterial identification and varying concentrations of antimicrobial agents on an AST side. To optimize testing performance, the tested isolates were incubated overnight at 35 °C on yeast extract agar (Merck, Germany). Afterwards, an initial suspension was prepared using Phoenix-ID broth, adjusted to a density of 0.5 MacFarland. Next, 25 μl of the initial inoculum was transferred into Phoenix-AST broth. Final suspensions of ID broth and AST broth were used for the inoculation of BD panels. Once inoculated, the panels were placed into the Phoenix Automated Microbiology System and incubated at 35 °C. Then, the identification was evaluated according to the presence/absence of colorimetric and fluorometric reactions, while organism growth in the presence/absence of an antimicrobial agent (turbidimetry and redox reactions) was used to determine the minimum inhibitory concentration (MIC). MICs obtained with the Phoenix system were then estimated using European Committee on Antimicrobial Susceptibility Testing breakpoints (EUCAST 2015). The isolates resistant to three or more antimicrobial classes, acc. to Magiorakos et al. (2012), were regarded as MDRs (Table 1). Only acquired antimicrobial resistance was taken into consideration in creating definitions for MDR (intrinsic resistance was not addressed). The quality control was performed using *P. aeruginosa* ATCC 27853 in each experimental set.

**Table 1:** Antimicrobial categories used to define MDR with the MIC interpretive standards used to define the susceptibility of *Pseudomonas* spp. to tested antimicrobial agents

Antimicrobial			MIC breakpoint^1^ mg L^−1^	
Category^2^	Agent	Code	S ≤	R >
Antimicrobial categories used to define MDR				
Aminoglycosides	Amikacin	AN	8	16
	Gentamicin	GM	4	4
	Tobramycin	NN	4	4
Antipseudomonal carbapenems	Imipenem	IMP	4	8
	Meropenem	MEM	2	8
Antipseudomonal cephalosporins	Ceftazidime	CAZ	8	8
	Cefepime	FEP	8	8
Antipseudomonal fluoroquinolones	Ciprofloxacin	CIP	0.5	1
Antipseudomonal penicillins + β-lactamase inhibitors	Ticarcillin-clavulanic acid	TIM	16	16
	Piperacillin-tazobactam	TZP	16	16
Monobactams	Aztreonam	ATM	1	16
Polymyxins	Colistin	CL	4	4
Other antimicrobial categories				
Antipseudomonal penicillins	Ticarcillin	TIC	16	16
	Piperacillin	PIP	16	16

^1^According to EUCAST (2015)

^2^According to Magiorakos et al. (2012)

### Sequencing and phylogenetic analysis

*Pseudomonas* spp. of raw (INF) and treated (EFF) wastewater as well as of marine water origin (MOut) were identified by biochemical methods. Those, identified only to the genera level, were additionally subjected to sequencing of 16S rDNA gene using primers F27 and R1492 (Lane 1991). Sequencing was also performed to confirm the identification of four selected isolates obtained from MOut and identified to species level (*P. aeruginosa*, *P. fluorescens*, and two isolates of *P. pseudoalcaligenes*). Prior sequencing DNA was extracted using boiling of several bacterial colonies in a water bath (5 min.). Cell debris was removed by centrifugation (11,357*g*, 5 min., Eppendorf MiniSpin Plus centrifuge, Germany), and the resulting supernatant was used as a template for PCR. All PCR reactions were carried out in a T1 thermal cycler (Biometra, Germany) using nucleotides, buffers, and *Taq* polymerase purchased from A&A Biotechnology (Poland), and sequencing was done using ABI PRISM BigDye Terminator cycle sequencing (Macrogen, Korea). Obtained sequences were compared to sequences in the GenBank database using the blastn algoritm (http://blast.ncbi.nlm.nih.gov) (Altschul et al. 1990). The 16S rRNA gene sequences obtained in this work were submitted to GenBank under accession numbers: KP763815-KP763830.

The sequences of 16S rRNA gene from isolates were aligned against the reference sequences from the GenBank database (*P. fluorescens* AB680973, *P. grimontii* NR025102, *P. mandelii* NR024902, *P. protegens* NR074599, *P. anguilliseptica* NR029319, *P. aeruginosa* NR074828, *P. stutzeri* NR118798, and *P. pseudoalcaligenes* NR118798). A phylogenetic tree was constructed from the alignment using the MEGA6 program (Tamura et al. 2013) by applying the neighbor-joining method. Bootstrapping was performed using 1000 replicates.

### Statistical analysis

Data used for statistical analyses was in 0/1 format with 1 denoting the occurrence of resistance to antimicrobial agent considered; whereas, 0 denotes susceptibility. Due to the data format, only methods applicable to contingency tables, such as Fisher exact test (Weisstein 2014) or test for homogeneity of proportions, were valid for use. Statistical analyses were performed to find dependencies between the antimicrobial resistance patterns between pairs of microbial agents. Additionally among *P. putida*, the dependencies of prevalence of resistance (especially MDR) and the point of sample collection were tested. The proportions of resistant and susceptible *Pseudomonas* spp. among those which were resistant to certain antibiotic were also tested.

## Results

Over the whole study period, the selected physical and chemical parameters as well as the *Pseudomonas* spp. load were analyzed in the influent (INF), effluent (EFF), and the marine outfall (MOut) of the wastewater treatment plant. The obtained results are listed in Table 2. The total *Pseudomonas* count depended on the sampling points, and the highest number was detected in raw wastewater.

**Table 2:** Physicochemical parameters and the presence of *Pseudomonas* spp. in wastewater and marine water samples (min–max)

Parameter	INF	EFF	MOut
BOD [mg 0_2_/dm^3^]	410–560	3–10	2–4
COD [mg 0_2_/dm^3^]	1200–1300	21.2–42.5	9.2–12.8
TN [mg N/dm^3^]	82.5–92.2	5.6–9.9	<0.001
TP [mg P/dm^3^]	10.4–13.2	0.45–0.96	<0.05–0.019
Total suspended solids [mg/dm^3^]	350–475	7.4–23.2	2.3–5.4
*Pseudomonas* spp. [CFU/100 mL]	3.1 × 10^5^–8.9 × 10^5^	1.3 × 10^4^–3.8 × 10^5^	0.2 × 10^2^–3.1 × 10^3^

Abbreviations of sampling points’ locations provided in Fig. 1

### Identification of the isolates

**Fig. 2:**
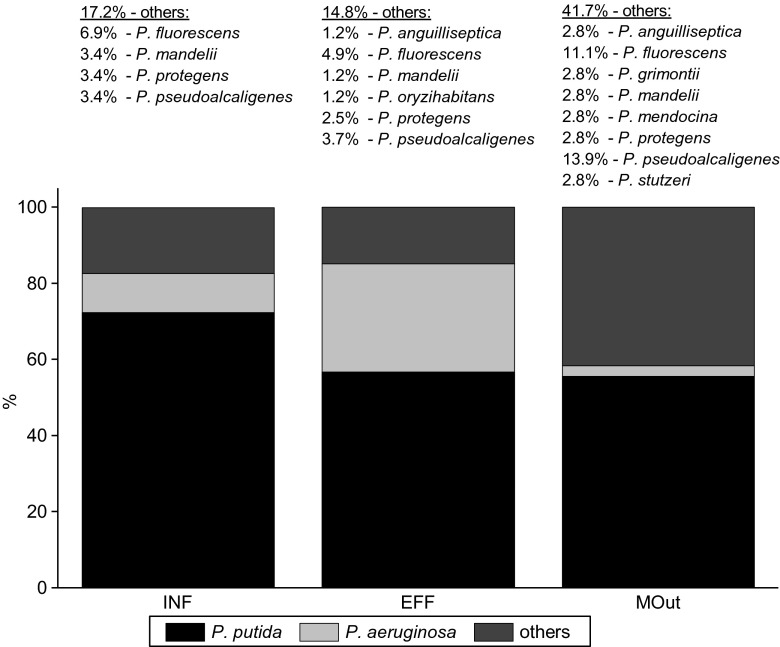
Distribution of *Pseudomonas* spp. in influent (*INF*), effluent (*EFF*), and marine outfall (*MOut*) of WWTP

**Fig. 3:**
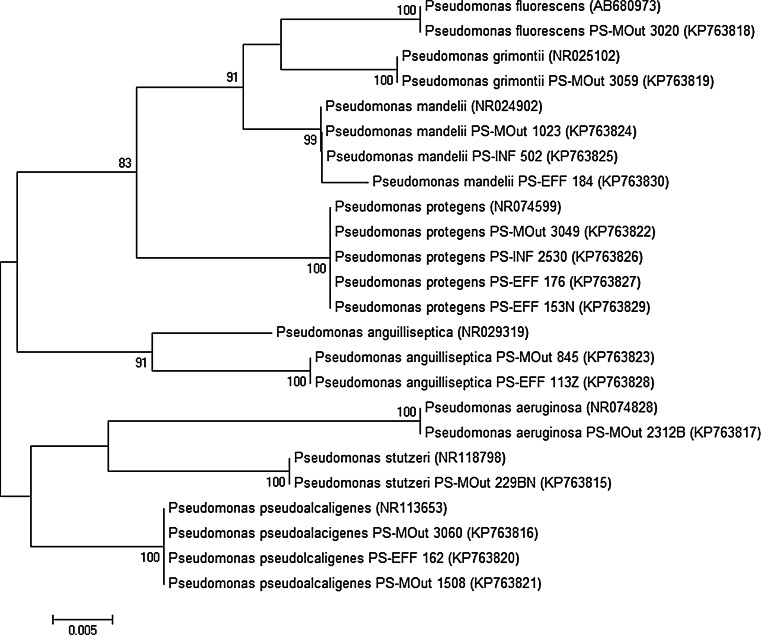
Neighbor-joining phylogenetic tree based on 16S rRNA gene sequences showing the relationships of 16 (selected) isolates with representative members of the genus *Pseudomonas*. The numbers after each isolate are the accession numbers of sequences in the GenBank database. *Numbers* indicated bootstrap percentages (based on 1000 replications). Bootstrap values >70 % are shown at *branch points. Bar*, 0.05 substitutions per nucleotide position

The 146 presumptive isolates of *Pseudomonas* spp. were analyzed in this study, 29 from INF, 81 from EFF, and 36 from MOut. Among them, 91.8 % of the isolates (134/146) were identified to genus level, while the remaining only to the genera level (8.2 %, 12/146). These isolates (two from INF, five from EFF, and five from MOut) were additionally subjected to the sequencing of the 16S rDNA gene. Based on 16S rDNA gene similarities (99 or 100 % similarity to reference sequences from the GenBank database), *Pseudomonas* spp. of INF origin were classified as *P. protegens* and *P. mandelii*; of EFF origin as *P. pseudoalcaligenes*, *P. anguilliseptica*, *P. mandelii*, and two *P. protegens*; while of MOut origin as *P. mandelii*, *P. protegens*, *P. pseudoalcaligenes*, *P. stutzeri*, *P. grimontii*, and *P. anguilliseptica*. Those results served as a final identification to the species level and were used throughout the study (Fig. 2). Additionally, sequencing confirmed the identification of four isolates obtained from marine water: *P. aeruginosa*, *P. fluorescens*, and two isolates of *P. pseudoalcaligenes*. The relationships among the 16 isolates and representatives of the genus *Pseudomonas* are shown in Fig. 3.

According to the obtained results, the tested isolates were mainly identified as *P. putida* (59.6 %, 87/146), followed by *P. aeruginosa* (18.5 %, 27/146). *P. putida* was predominant in all sampling points, from 72 % (21/29) in INF to 56 % (20/36) in MOut (Fig. 2), while *P. aeruginosa* consisted of 10.3 % in INF (3/29), 28.4 % in EFF (23/81), and 2.8 % in MOut (1/36). Additionally, 6.8 % of the isolates were identified as *P. fluorescens* (10/146), 6.2 % as *P. pseudoalcaligenes* (9/146), 2.7 % as *P. protegens* (4/146), and 2.1 % as *P. mandelii* (3/146). Single isolates of *P. oryzihabitans* as well as *P. stutzeri*, *P. grimontii*, and *P. mendocina* were detected in EFF and MOut samples, respectively (Fig. 2).

### Antimicrobial susceptibility profile

**Fig. 4:**
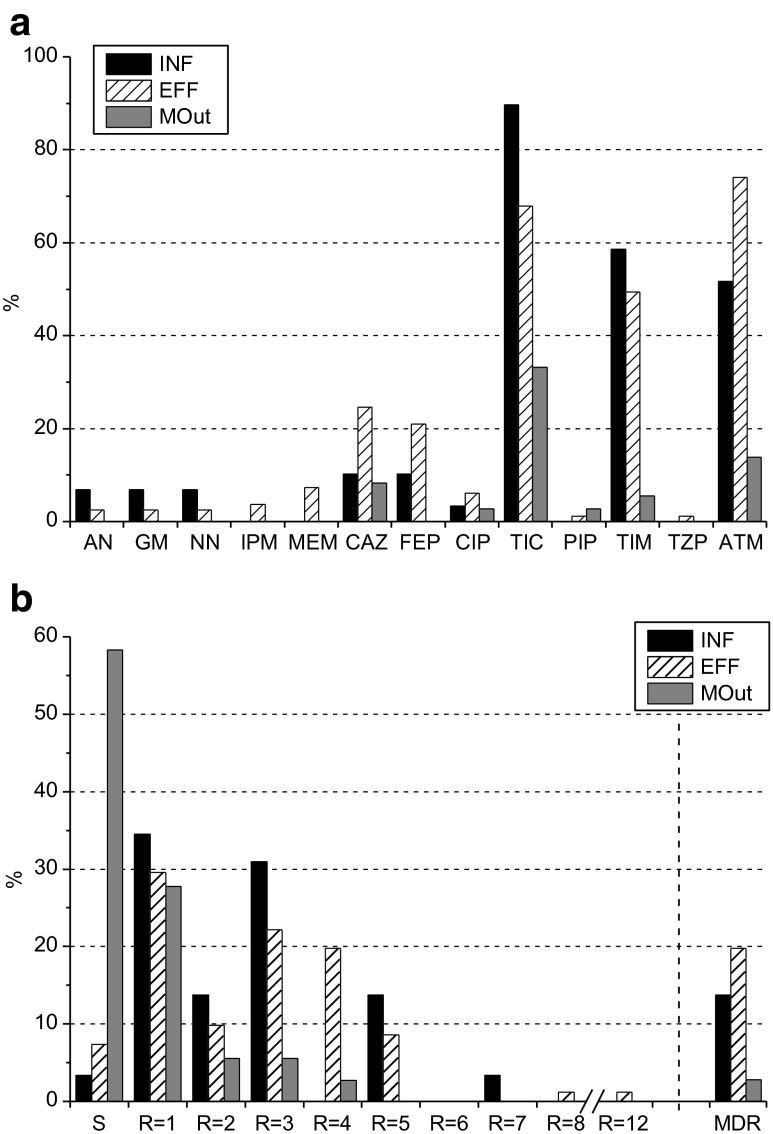
Antimicrobial resistance of *Pseudomonas* spp. **a** to the single antimicrobial agent, **b** to all tested antimicrobial agents (*S*—sensitive, *R* = *X*—resistant to X antimicrobial agents, *MDR*—multidrug resistant)

The level of resistance among *Pseudomonas* spp. was assessed against 14 antimicrobial agents. The general susceptibility of the genera will be reported for 146 isolates (Fig. 4), but the detailed only for predominant *P. aeruginosa* (27/146) and *P. putida* (87/146). In the case of the remaining species (32/146), their resistance rates cannot be counted as a representative, due to the very low number of isolates.

According to the obtained results, the resistance to colistin was not observed among *Pseudomonas* spp., either in the wastewater or marine water samples, but the majority of isolates showed resistance to aztreonam (54.8 %, 80/146) and ticarcillin (63.7 %, 93/146), especially in wastewater samples (Fig. 4). Resistance to a combination of ticarcillin with the β-lactamase inhibitor—clavulanate, was also commonly observed (40.4 %, 59/146). In the case of piperacillin, resistance was detected only in one isolate of *P. putida* of treated wastewater origin. This isolate was also the only one resistant to piperacillin—tazobactam. Resistance to tested aminoglycosides (amikacin, gentamicin, and tobramycin), carbapenems (imipenem and meropenem), and fluoroquinolones (ciprofloxacin) was also observed only among *P. putida* isolates (Table 3).

**Table 3:**
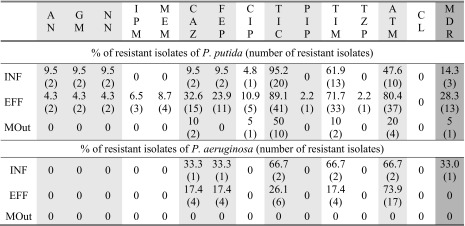
Susceptibility of *P. putida* and *P. aeruginosa*

Antimicrobial classes are distinguished by shading

*AN* amikacin, *GM* gentamicin, *NN* tobramycin, *IPM* imipenem, *MEM* meropenem, *CAZ* ceftazidime, *FEP* cefepime, *CIP* ciprofloxacin, *TIC* ticarcillin, *PIP* piperacillin, *TIM* ticarcillin-clavulanic acid, *TZP* piperacillin-tazobactam, *ATM* aztreonam, *CL* colistin, *MDR* multidrug resistance

Resistance to cefepime (4th generation cephalosporin) was detected in raw and treated wastewater for 9.5 % (2/21) and 23.9 % (11/46) isolates of *P. putida* and 33.3 % (1/3) and 17.4 % (4/23) isolates of *P. aeruginosa*, respectively. Similar resistance rates among *P. putida* and *P. aeruginosa* were obtained for 3rd generation cephalosporin, although two ceftazidime-resistant isolates of *P. putida* and one of *P. fluorescens* were additionally detected in MOut.

MDR patterns were detected in all sampling points (Fig. 4), mainly among *P. putida* (19.5 %, 17/87) and in one isolate of *P. aeruginosa* obtained from INF (Tables 3 and 4). Among other species, the MDR phenotype was detected only in treated wastewater, among three isolates: two *P. pseudoalcaligenes* and one confirmed by sequencing as *P. protegens* (Table 4). The dependency between the ratio of MDR *P. putida* and sample collection point turned out to be significant (*p* < 0.01).

**Table 4:**
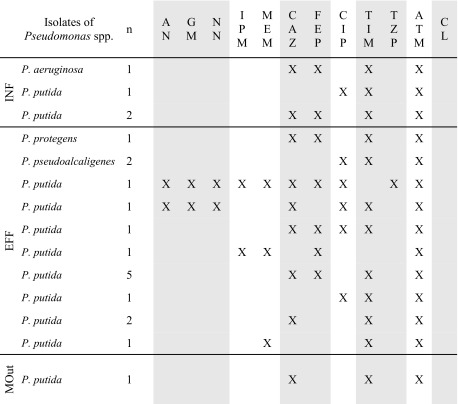
MDR phenotypes detected among *Pseudomonas* spp.

Antimicrobial classes are distinguished by shading

*AN* amikacin, *GM* gentamicin, *NN* tobramycin, *IPM* imipenem, *MEM* meropenem, *CAZ* ceftazidime, *FEP* cefepime, *CIP* ciprofloxacin, *TIC* ticarcillin, *PIP* piperacillin, *TIM* ticarcillin-clavulanic acid, *TZP* piperacillin-tazobactam, *ATM* aztreonam, *CL* colistin, *MDR* multidrug resistance

## Discussion

*Pseudomonas* spp. due to their functional versatility are usually active in different natural and artificial niches (Mercier and Lindow 2000; Moore et al. 2006; Vaz-Moreira et al. 2012). An increasing interest is given to *Pseudomonas* spp. as etiological agents of numerous hospital-associated infections as well as their application in bioremediation and plant protection. Biofilm formation, besides the diverse metabolism, is an important feature of pseudomonades, which enables them to colonize different surfaces and ensure their survival in adverse and fluctuating conditions (Singh et al. 2000; Carpenter et al. 2008). *Pseudomonas* spp. can colonize various hospital niches, are also present in water systems (Kelsey 2013), and are detected as active members of activated sludge, in both municipal and industrial wastewater treatment plants (Schwartz et al. 2006; Moura et al. 2010).

In this study, the presence and distribution of cultivable *Pseudomonas* spp. were analyzed in the samples of raw and treated wastewater as well as its receiver—coastal waters of the Puck Bay, Baltic Sea. The *Pseudomonas* spp. isolated in this study comprise 12 species: *P. putida*, *P. aeruginosa*, *P. fluorescens*, *P. pseudoalcaligenes*, *P. protegens*, *P. mandelii*, *P. anguilliseptica*, *P. grimontii*, *P. mendocina*, *P. oryzihabitans*, and *P. stutzeri* (Fig. 2). Among them, *P. aeruginosa* is regarded as an opportunistic pathogen that infects humans with compromised natural defense, mainly in hospital units. But also *P. putida*, *P. fluorescens*, *P. mendocina*, *P. oryzihabitans*, and *P. stutzeri* are occasionally recovered from some human clinical samples and are linked to opportunistic nosocomial infections of immunocompromised patients (Hsueh et al. 1998; Yoshino et al. 2011). *Pseudomonas* spp. can cause animal- and plant-associated diseases. Among the detected species, *P. aeruginosa*, *P. fluorescens*, and *P. stutzeri* can infect birds, while *P. anguilliseptica*, *P. fluorescens*, and *P. pseudoalcaligenes* can infect fishes. But besides pathogenicity, *P. putida* and *P. fluorescens* were recognized as rhizosphere colonizers with the potential to contribute to plant growth and to plant disease resistance.

According to the obtained results, *P. putida* was the predominant microorganism in both raw and treated wastewater as well as in marine outfall (Fig. 2), while the relative number of *P. aeruginosa* was the highest in EFF comparing to INF and MOut (Fig. 2). The distribution of *Pseudomonas* spp. in different environments is not well understood. In tested niches, the highest species diversity was obtained in MOut (Fig. 2).

In general, a remarkable adaptability of *Pseudomonas* spp. is usually linked to the enhanced evolvability (Spiers et al. 2000) and ability to develop resistance by horizontal gene transfer (Carmeli et al. 1999). In this study, the susceptibility to antimicrobial agents active against *Pseudomonas* infections was evaluated according to the rules defined by EUCAST (2015), excluding the antimicrobial agents to which *P. aeruginosa* is intrinsically resistant (Leclercq et al. 2013); thus, obtained results can be compared with data of epidemiological surveillance systems (EARS-Net 2013).

Among tested *Pseudomonas*, most isolates (80.8 %, 118/146) were found to be resistant at least to one of the tested antimicrobial agents (Fig. 4b). A significant difference of resistance rates was observed among tested sampling points. In raw (INF) and treated wastewater (EFF), over 90 % of the isolates were resistant (28/29 and 75/81, respectively), while the resistance rate decreased significantly in MOut (41.7 %, 15/36).

The resistance to piperacillin as well as to the combination of piperacillin with tazobactam was observed rarely (one *P. putida* in EFF and one *P. pseudoalcaligenes* in MOut). It is mildly surprising since in Poland, resistance to piperacillin reached 30 % in 2012 among invasive *P. aeruginosa* (EARS-Net 2013).

Resistance to extended spectrum penicillin—ticarcillin, particularly combined with a β-lactamase inhibitor—clavulanic acid, was commonly observed in all sampling points (Fig. 4), which is in line with expectations since ticarcillin is usually used in this combination.

Resistance to carbapenems (meropenem and imipenem), clinically relevant β-lactamases with the widest spectrum of activity, was observed relatively rarely, only in treated wastewater. Carbapenems are considered first-line agents in treating infections caused by ESBL-producing organisms. In Poland, however, the infections caused by ESBL-producing *P. aeruginosa* were rarely reported (Empel et al. 2007). Clinical data suggested that extended-spectrum cephalosporinases probably contribute to carbapenem resistance (Rodríguez-Martínez et al. 2009). Among tested *Pseudomonas* spp. resistant to extended-spectrum cephalosporins (ceftazidime and cefepime), no significant difference (*p* < 0.01) of proportions between resistant and susceptible to carbapenems was found. In Poland, clinical isolates of *P. aeruginosa* showed similar resistance rate to carbapenems and ceftazidime, reaching 23 and 24 %, respectively in 2012 (EARS-Net 2013).

β-Lactams mentioned above are often used in the treatment of severe pseudomonad infection; however, it is suggested that monotherapy is less effective than the combination of β-lactams with aminoglycosides or fluoroquinolones (Livermore 2002). In this study, only *P. putida* showed resistance against aminoglycosides: 9.5 % isolates in INF (2/21) and 4.3 % in EFF (2/46) (Table 3).

The resistance to ciprofloxacin (a second-generation fluoroquinolone) was detected among *P. putida* (INF—4.8 %, 1/21, EFF—10.9 %, 5/46; MOut—5 %, 1/20) as well as among *P. pseudoalcaligenes* in EFF (33.3 %, 1/3). All fluoroquinolones are effective against Gram-negative bacteria, although ciprofloxacin was suggested to be the most effective against *P. aeruginosa*. Invasive isolates of *P. aeruginosa* from Polish care units were found to be 27 % resistant to fluoroquinolones in 2012 (EARS-Net 2013).

In the case of colistin, all tested isolates were susceptible to this antimicrobial agent. Also in clinical reports, colistin-resistant *Pseudomonas* spp. are reported rarely. This cyclic cationic peptide disturbs bacterial outer membrane permeability by interacting with lipopolysaccharide and phospholipids; therefore, it is active against Gram-negative bacteria. But usage of colistin is limited only to the patients infected by multidrug-resistant strains, especially *Pseudomonas*, *Acinetobacter*, and *Stenotrophomonas*, due to its nephro- and neurotoxicity (Sabuda et al. 2008). Also, the exact mechanisms of bacterial resistance against colistin are not clear (Lim et al. 2010).

Aside from resistance to a single antimicrobial agent, the most challenging public health problem is the increasing number of MDR bacteria. The real prevalence of MDRs is, however, not well established, due to usage of different definitions in literature. Only since 2012, the standardized international MDR terminology has been introduced by the European Centre for Disease Prevention and Control (ECDC) and the Centers for Disease Control and Prevention (CDC) for bacteria (including *P. aeruginosa*) linked to healthcare-associated infections (Magiorakos et al. 2012). This terminology was also used in the present study; thus, in MDR evaluation, resistance to piperacillin and ticarcillin was not considered (Table 1). According to the obtained results, MDR *Pseudomonas* spp. occurred in INF, EFF, and MOut with varying prevalence, equal 13.8 % (4/29), 19.8 % (16/81), and 2.8 % (1/36), respectively. MDR patterns were detected mainly among *P. putida* (19.5 %, 17/87), two identified as *P. pseudoalcaligenes*, and one as *P. protegens* (Tables 3 and 4).

EARS-Net (2013) reports MDR patterns among invasive isolates of *P. aeruginosa*, tested against piperacillin-tazobactam, ceftazidime, fluoroquinolones, aminoglycosides, and carbapenems. According to this report, most common 3-classes MDRs were detected against fluoroquinolones + aminoglycosides + carbapenems while 4-classes MDR against piperacillin-tazobactam + fluoroquinolones + aminoglycosides + carbapenems. The MDR patterns obtained in this study are listed in Table 4 and presented in Fig. 4. Usually, MDR isolates showed resistance against antimicrobials from the three of tested classes: cephalosporins (ceftazidime and cefepime), penicillins with β-lactamase inhibitors (ticarcillin with clavulanic acid), and monobactams (aztreonam). One of all the tested isolates, originated from treated wastewater and identified as *P. putida*, was resistant to all tested classes but polymyxins (colistin). Additionally, resistance to four classes was observed in two strains of *P. putida*, in one isolated from INF and one isolated from EFF. A significant correlation was detected between resistance to aztreonam and the MDR profile, since all MDRs were aztreonam-resistant, regardless of the species and place of isolation.

Clinical data indicated that *P. aeruginosa* can develop resistance even during the course of medical treatment. This study also suggests that antimicrobial resistance may facilitate survival of *Pseudomonas* spp., since some phenotypes become more prevalent after wastewater treatment, and resistance prevalence was found to be significantly dependent on sampling point (*p* < 0.01). This phenomenon was mainly observed among *P. putida* resistant to tested carbapenems (meropenem and imipenem), cephalosporins (ceftazidime and cefepime), aztreonam, and ciprofloxacin as well as those showing the MDR patterns.

It is suggested that all stressors in wastewater, such as residues of antimicrobials, heavy metals, etc., may result in the selection of antibiotic-resistant bacteria (Zhang et al. 2011; Kotlarska et al. 2015) and positively affect transfer of resistance determinants (Inoue et al. 2005; Merlin et al. 2011). Antimicrobial resistance can be developed by mutation of existing genes, defined as vertical evolution, or by horizontal gene transfer that refers to the acquisition of new genes from other strains via mobile genetic elements (e.g., plasmids, transposons, or integrons). It has already been observed that the transfer of plasmids between different strains of *P. putida* can occur (Ashelford et al. 1995), but it is limited by nutrient availability and the physiological activity of the recipient. Thus, it is suggested that bacteria active in wastewater processes have high potential as plasmid recipients (Inoue et al. 2005). Additionally, the microbial density and diversity of activated sludge may facilitate the horizontal transfer of different genes, including resistance ones. The natural state of competence required to uptake the foreign genetic material in situ was confirmed among *P. fluorescens* (Demanèche et al. 2001) and *P. stutzeri* (Sikorski et al. 2002). Additionally, it was suggested that the members of *Pseudomonas* spp. were found to easily develop resistance to antimicrobials through the acquisition of plasmid-mediated resistance genes. Soda et al. (2008) indicated *P. putida* and *P. fluorescens* as dominant transconjugant strains among indigenous activated sludge bacteria. Among *Pseudomonas* spp. identified in this study, especially *P. putida* can be specifically suggested as a reservoir of antimicrobial resistance, which is in agreement with the results of Meireles et al. (2013). Also, Tsutsui et al. (2010) suggested that the plasmid pJP4 was preferentially transferred to members of *Pseudomonas* spp., although longer sludge retention time increased phenotypical diversity among activated sludge transconjugants. Sludge retention time was also suggested to influence resistance rate among enteric bacteria (Luczkiewicz et al. 2010). These findings indicated that the processes used to treat wastewater in studied WWTP may influence susceptibility of indigenous and non-indigenous bacteria of activated sludge.

In the absence of selective pressure, the resistance to antibiotics usually incurs a fitness cost, which is suggested as a key player in the spread of antibiotic resistance. The ecological fitness of antibiotic resistance (plasmid replication, efflux pumps, and others) must be compensated by facilitate, e.g., environmental survival. In these terms, the subinhibitory concentrations of antimicrobial agents are discussed as a kind of impact affecting resistance dissemination. The presence of antimicrobials and their residues in wastewater was reported in several studies (e.g., Kümmerer 2009; Luczkiewicz et al. 2013). In raw wastewater, it seems to be related to the local consumption rate and the form of antimicrobial excretion, while in treated wastewater to applied treatment technology (Göbel et al. 2007). Nonetheless, antimicrobial agents are found in wastewater and wastewater-impacted ecosystems (Kümmerer 2009). There is also evidence that concentrations of antimicrobial agents found in wastewater can influence the free living bacteria as well as biofilm structure in an unpredictable way (Bruchmann et al. 2013). In this study, dilution of treated wastewater by the submarine diffusers can, to some extent, explain observed decrease of the resistance rate among isolates of marine water origin. But the role of antimicrobials in wastewater and wastewater-impacted ecosystems remains a controversial issue.

## Conclusions

*Pseudomonas* spp. showed a high resistance rate in wastewater samples (over 90 % of isolates were resistant to at least one of tested antimicrobial agents). It suggests that *Pseudomonas* spp. are equipped with a wide range of antibiotic resistance mechanisms, especially *P. putida*, which was predominantly detected in this study. Increasing the resistance rate to all tested β-lactams and fluoroquinolones as well as increasing number of MDR isolates observed among *P. putida* during the wastewater course suggested *P. putida* as a potential recipient and reservoir of antimicrobial-resistance genes. However, the MDR phenotype was also detected among *P. aeruginosa*, *P. fluorescens*, *P. pseudoalcaligenes*, and *P. protegens*. Obtained data suggests that *Pseudomonas* spp. should be monitored as relevant source of resistance determinants, even though the resistance rate decreased significantly in marine water samples.
